# Canagliflozin mediated dual inhibition of mitochondrial glutamate dehydrogenase and complex I: an off-target adverse effect

**DOI:** 10.1038/s41419-018-0273-y

**Published:** 2018-02-14

**Authors:** Philipp F. Secker, Sascha Beneke, Nadja Schlichenmaier, Johannes Delp, Simon Gutbier, Marcel Leist, Daniel R. Dietrich

**Affiliations:** 1 Human and Environmental Toxicology, University of Konstanz, 78457 Konstanz, Germany; 2 In-vitro Toxicology and Biomedicine, University of Konstanz, 78457 Konstanz, Germany

## Abstract

Recent FDA Drug Safety Communications report an increased risk for acute kidney injury in patients treated with the gliflozin class of sodium/glucose co-transport inhibitors indicated for treatment of type 2 diabetes mellitus. To identify a potential rationale for the latter, we used an in vitro human renal proximal tubule epithelial cell model system (RPTEC/TERT1), physiologically representing human renal proximal tubule function. A targeted metabolomics approach, contrasting gliflozins to inhibitors of central carbon metabolism and mitochondrial function, revealed a double mode of action for canagliflozin, but not for its analogs dapagliflozin and empagliflozin. Canagliflozin inhibited the glutamate dehydrogenase (GDH) and mitochondrial electron transport chain (ETC) complex I at clinically relevant concentrations. This dual inhibition specifically prevented replenishment of tricarboxylic acid cycle metabolites by glutamine (anaplerosis) and thus altered amino acid pools by increasing compensatory transamination reactions. Consequently, canagliflozin caused a characteristic intracellular accumulation of glutamine, glutamate and alanine in confluent, quiescent RPTEC/TERT1. Canagliflozin, but none of the classical ETC inhibitors, induced cytotoxicity at particularly low concentrations in proliferating RPTEC/TERT1, serving as model for proximal tubule regeneration in situ. This finding is testimony of the strong dependence of proliferating cells on glutamine anaplerosis via GDH. Our discovery of canagliflozin-mediated simultaneous inhibition of GDH and ETC complex I in renal cells at clinically relevant concentrations, and their particular susceptibility to necrotic cell death during proliferation, provides a mechanistic rationale for the adverse effects observed especially in patients with preexisting chronic kidney disease or previous kidney injury characterized by sustained regenerative tubular epithelial cell proliferation.

## Introduction

Canagliflozin is a member of the gliflozin group of pharmaceuticals indicated for treatment of type 2 diabetes mellitus (T2DM). Gliflozins are inhibitors of members of the sodium-coupled glucose co-transporters (SGLT; *SLC5A* gene family)^1^ and primarily target SGLT2 expressed in renal proximal tubule epithelial cells (RPTECs) of the kidney. SGLT2 is responsible for the bulk of renal glucose reabsorption, while the SGLT1 isoform, expressed in the pars recta of the renal proximal tubule, is a high-affinity/low-capacity transporter, responsible for the uptake of the remaining glucose and galactose molecules in the primary urine. SGLT1 is also expressed in the brush border membrane of the small intestine^2^. Two inherited human disorders of sodium-coupled glucose transport, i.e., intestinal glucose-galactose malabsorption (GGM), involving SGLT1 gene mutations, and familial renal glucosuria (FRG), involving mutations of the SGLT2 gene, are known to date. Neither GGM nor FRG disorders are accompanied by serious health issues for the affected individuals, nor have they been specifically associated with intestinal or renal pathology^2^. Hence, the inhibition of renal SGLT2 was considered useful for treatment of T2DM, which was supported by studies with the natural compound phlorizin, a metabolically unstable and unspecific inhibitor of SGLT2 and SGLT1^3^. Accordingly, analogs of phlorizin, yet with higher selectivity of SGLT2 over SGLT1^4^ and increased stability and bioavailability, were developed to increase urinary clearance of blood glucose. Three such SGLT2 inhibitors, canagliflozin (Invokana®), dapagliflozin (Forxiga®) and empagliflozin (Jardiance®), are currently approved by the Food and Drug Administration (FDA) and the European Medicines Agency (EMA) for treatment of T2DM. The pharmacology of SGLT2 inhibition is generally regarded as safe, mainly because of the low risk of hypoglycemia and in conjunction with the benign conditions of GGM and FRG patients. However, recent FDA Drug Safety Communications do suggest that canagliflozin, and to a lesser extent dapagliflozin, could be nephrotoxic in patients with preexisting chronic kidney disease or previous kidney injury^5^ and that gliflozin use is associated with an increased risk of diabetic ketoacidosis^6^.

Consequently, we compared the cytotoxicity of dapagliflozin, empagliflozin and canagliflozin in quiescent and proliferating human RPTEC/TERT1 cells and investigated the potential direct interference of gliflozins with RPTEC/TERT1 energy metabolism. RPTEC/TERT1 cells were derived from primary human RPTECs immortalized by transfection with telomerase^7^, which largely retained their expression profile and functionality^8,9^. Via cultivation for 10 days after reaching confluency, these cells can be converted to a differentiated cell monolayer^8^, displaying functional and morphological changes that mimick the healthy proximal tubule epithelium in situ. RPTEC/TERT1 cells cultured under proliferating conditions served as model for tubule epithelial cell regeneration^10^. We found that canagliflozin, but not dapagliflozin or empagliflozin, exhibited an off-target, and thus SGLT2-independent adverse effect, characterized by the dual inhibition of glutamate dehydrogenase (GDH) and complex I of the mitochondrial electron transport chain (ETC) at pharmacologically relevant concentrations. This combined ETC and GDH inhibition obstructed glutamine input into the tricarboxylic acid (TCA) cycle (i.e. glutamine anaplerosis). As proliferating cells are much more dependent on anaplerosis, this dual inhibition explains why canagliflozin is significantly more toxic for proliferating than for quiescent cells and considerably more potent than classical ETC inhibitors. Thus, our findings demonstrate that canagliflozin interferes with essential energy pathways in glutamine-dependent human cells. This offers a novel mechanistic explanation for the nephrotoxicity reported in patients with increased regenerative cell proliferation, e.g., observed subsequent to acute kidney injury or in chronic kidney disease.

## Results

### Canagliflozin increased aerobic glycolysis and triggered cytotoxicity in RPTEC/TERT1 cells

**Fig. 1:**
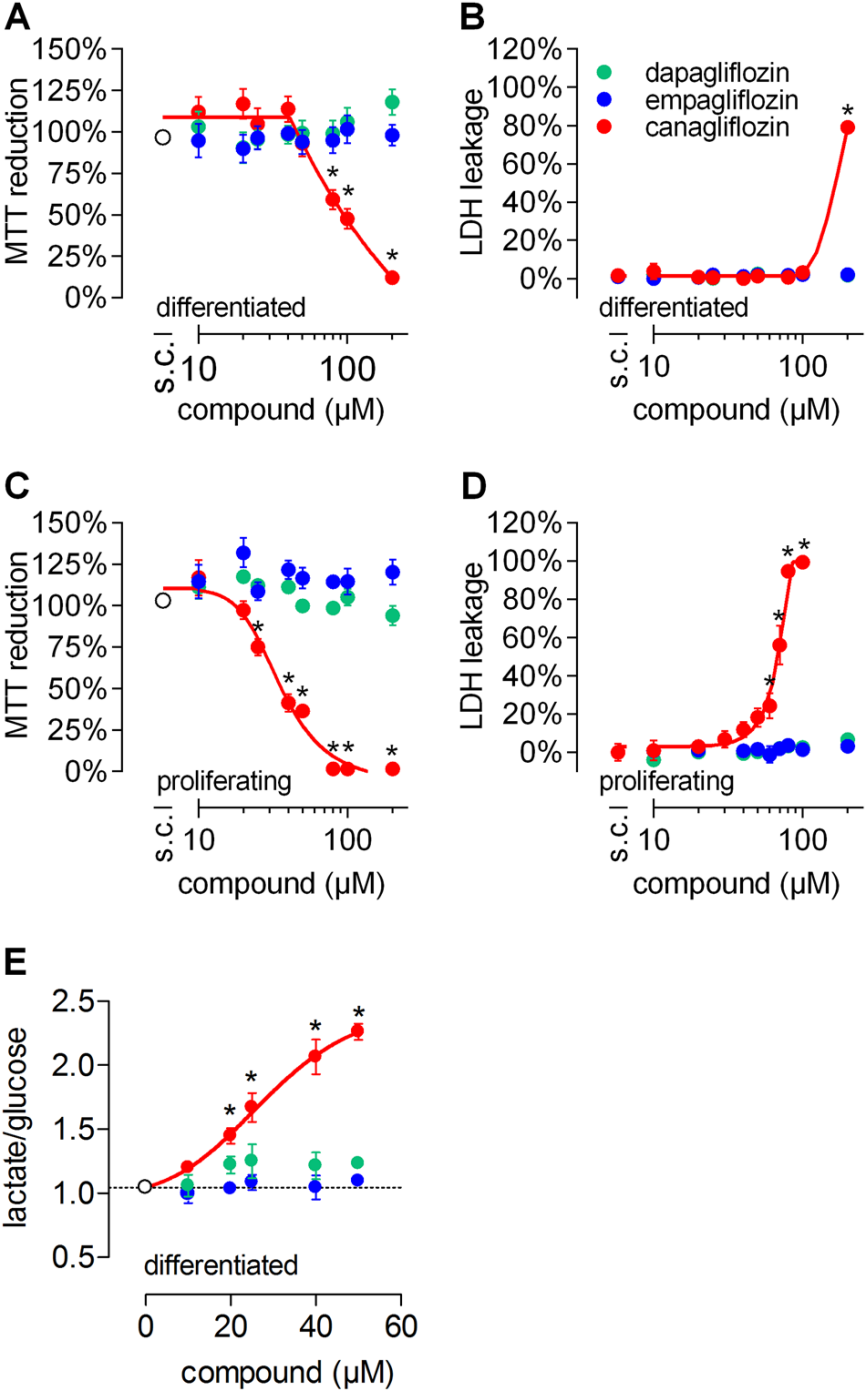
Canagliflozin is cytotoxic for RPTEC/TERT1 cells and causes aerobic glycolysis at subtoxic concentrations. **a**, **b**, **e** Differentiated (day 16) or **c**, **d** proliferating RPTEC/TERT1 cells were exposed to the different gliflozins in the presence of 0.5% DMSO for 24 h followed by quantification of **a**, **c** cell viability via MTT reduction or **b**, **d** cytotoxicity via LDH leakage. MTT reduction is normalized to non-treated cells. **e** Ratio of secreted lactate divided by consumed glucose in differentiated RPTEC/TERT1 cells as indicator of aerobic glycolysis following 24 h of exposure to gliflozins at subtoxic concentrations. Data are mean ± SEM of at least three independent experiments performed in duplicate. *P < 0.05 relative to 0.5% DMSO (solvent control; s.c.) assessed by one-way analysis of variance (ANOVA) with Dunnett’s post-test

RPTEC/TERT1 cells can be cultured to a contact-inhibited monolayer reflecting primary RPTECs^7,8^, from here on referred to as differentiated RPTEC/TERT1. We exposed proliferating (day 1 after seeding) and differentiated cells (day 16 after seeding) for 24 h to canagliflozin, dapagliflozin or empagliflozin at concentrations reflecting those observed in clinical and preclinical (rodent) settings^11–16^. Subsequently, 3-(4,5-dimethylthiazol-2-yl)-2,5-diphenyltetrazolium bromide (MTT) reduction (cell viability) and lactate dehydrogenase (LDH) leakage (cytotoxicity) was quantified. Of the gliflozins tested, only canagliflozin showed cytotoxicity (Fig. 1a–d), and was more cytotoxic in proliferating than in quiescent cells (Fig. 1c, d). Necrotic cell death was confirmed by morphological changes and absence of annexin V staining (Fig. S1A). Canagliflozin did not induce autophagy as shown via lysosomal compartment staining with LysoID and immunoblots of LC3B conversion (Fig. S1B). Since the MTT assay depends on cellular redox status maintained by mitochondrial activity, we analyzed the cellular glycolytic rate, commonly increased when mitochondrial function is impaired. In differentiated cells, canagliflozin, but not the other two gliflozins, significantly raised cellular glucose to lactate conversion (Fig. 1e). Moreover, canagliflozin increased both total lactate secretion (Fig. S2A) and glucose consumption (Fig. S2B), a characteristic response observed with mitochondrial impairment.

### Canagliflozin specifically inhibited ETC complex I

**Fig. 2:**
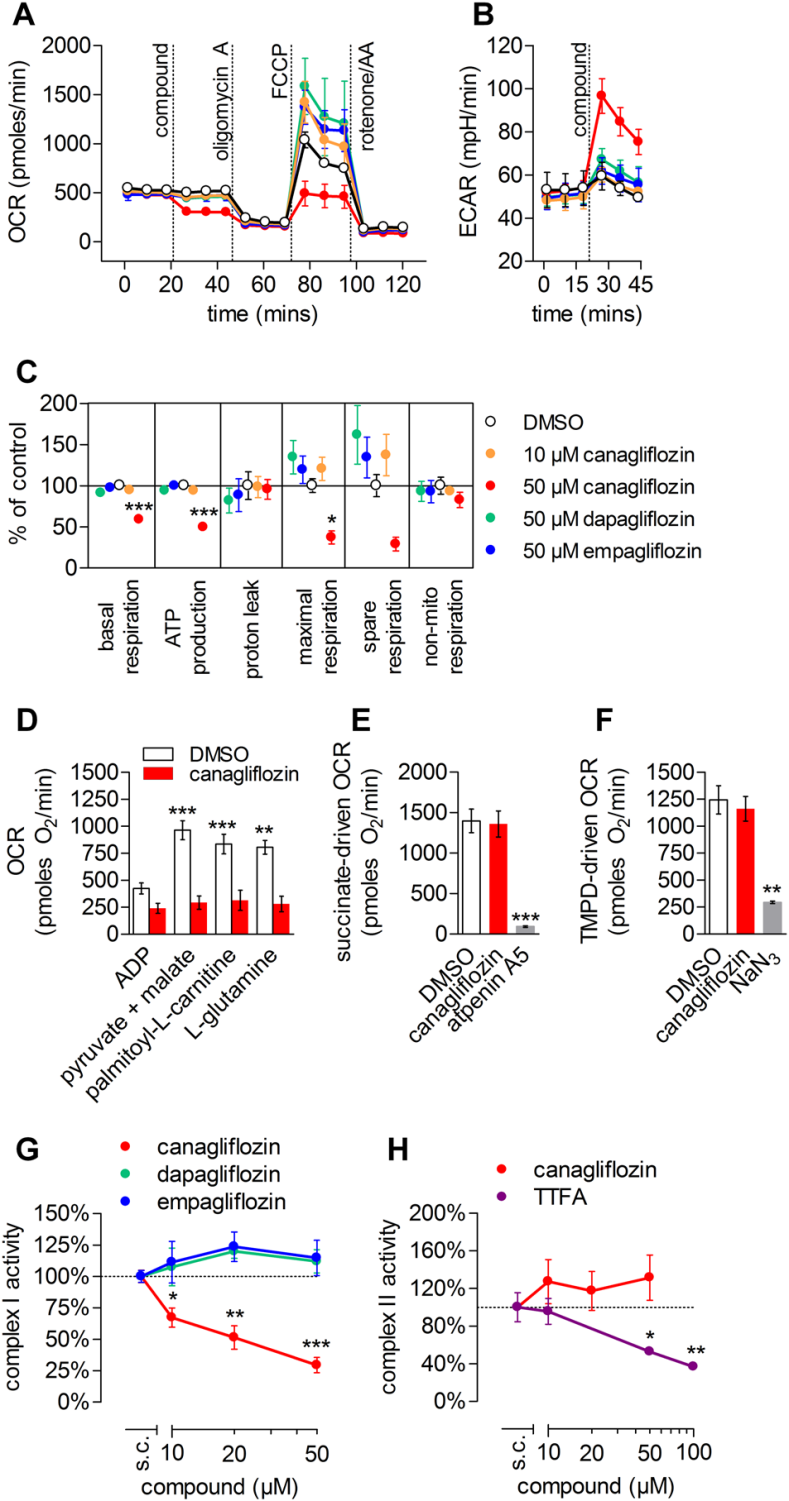
Canagliflozin interferes with the ETC by inhibiting complex I. **a** Oxygen consumption rate (OCR) and **b** extracellular acidification rate (ECAR) of a Seahorse Mito Stress Profile using differentiated RPTEC/TERT1 acutely exposed to the different gliflozins. Mean ± SD of a representative experiment performed in triplicate. **c** Key parameters of mitochondrial respiration. Mean ± SEM, *n* = 3 independent experiments performed in triplicate. *P < 0.05, ***P < 0.001 relative to 0.5% DMSO-treated assessed by one-way ANOVA with Bonferroni’s post-test. **d** Complex I-dependent respiration in permeabilized RPTEC/TERT1 cells exposed to 0.5% DMSO or 50 µM canagliflozin in the presence of either ADP (1 mM) alone or ADP with pyruvate (5 mM) and malate (2.5 mM), palmitoyl-L-carnitine (50 µM with 0.5 mM malate) or l-glutamine (4 mM with 0.5 mM malate). Mean ± SEM, *n* = 3 independent experiments. **P < 0.01, ***P < 0.001 relative to respective ADP only condition assessed by one-way ANOVA with Bonferroni’s post-test. **e** Complex II (10 mM succinate, 1 µM rotenone, 1 mM ADP) and **f** complex IV (1 µM rotenone, 1 µM antimycin A, 0.5 mM TMPD and 2 mM ascorbate) dependent respiration in permeabilized RPTEC/TERT1 cells exposed to 0.5% DMSO, 50 µM canagliflozin, 1 µM atpenin A5 or 10 mM NaN_3_. Mean ± SEM, *n* = 3 independent experiments performed in duplicate. **P < 0.01, ***P < 0.001 relative to respective 0.5% DMSO-treated cells assessed by one-way ANOVA with Bonferroni’s post-test. **g** Complex I and **h** complex II activity in mitochondrial extracts exposed to the different gliflozins or the complex II inhibitor TTFA. Mean ± SEM, *n* = 3 independent experiments. *P < 0.05, **P < 0.01, ***P < 0.001 relative to solvent control (s.c., 0.5% DMSO) assessed by one-way ANOVA with Dunnett’s post-test

Increased glucose to lactate conversion is often the consequence of a blocked mitochondrial electron transport chain^17,18^. To better characterize the presumed canagliflozin-induced impairment of mitochondrial function, we measured cellular oxygen consumption rates (OCR) and extracellular acidification rates (ECAR) of gliflozin-treated differentiated RPTEC/TERT1 cells (Fig. 2a, b). Canagliflozin at 50 µM, but not at 10 µM, significantly decreased basal respiration, mitochondrial adenosine triphosphate (ATP) production, maximal and spare respiration, but not proton leak and non-mitochondrial respiration (Fig. 2c). Concomitantly, ECAR was increased, suggestive of a direct glycolytic response (Fig. 2b). Equimolar dapagliflozin or empagliflozin had no effect. Plasma membrane permeabilization with digitonin allowed for delivery of specific metabolic substrates while maintaining mitochondrial integrity (Fig. S3). Accordingly, we determined that canagliflozin completely blocked mitochondrial respiration of the complex I substrates pyruvate, fatty acids and glutamine (Fig. 2d), but did not affect respiration from complex II and IV (Fig. 2e, f). To corroborate complex I-specific inhibition by canagliflozin, we prepared mitochondrial-enriched fractions of RPTEC/TERT1 cells and quantified rotenone-sensitive NADH oxidation (equivalent to complex I activity) as well as atpenin A5-sensitive succinate oxidation (equivalent to complex II activity). As expected, canagliflozin concentration-dependently reduced NADH oxidation (Fig. 2g) but did not affect succinate oxidation (Fig. 2h), thereby confirming canagliflozin-mediated impairment of the mitochondrial ETC via complex I inhibition.

### Canagliflozin generated a unique metabolic profile of amino acid accumulation

**Fig. 3:**
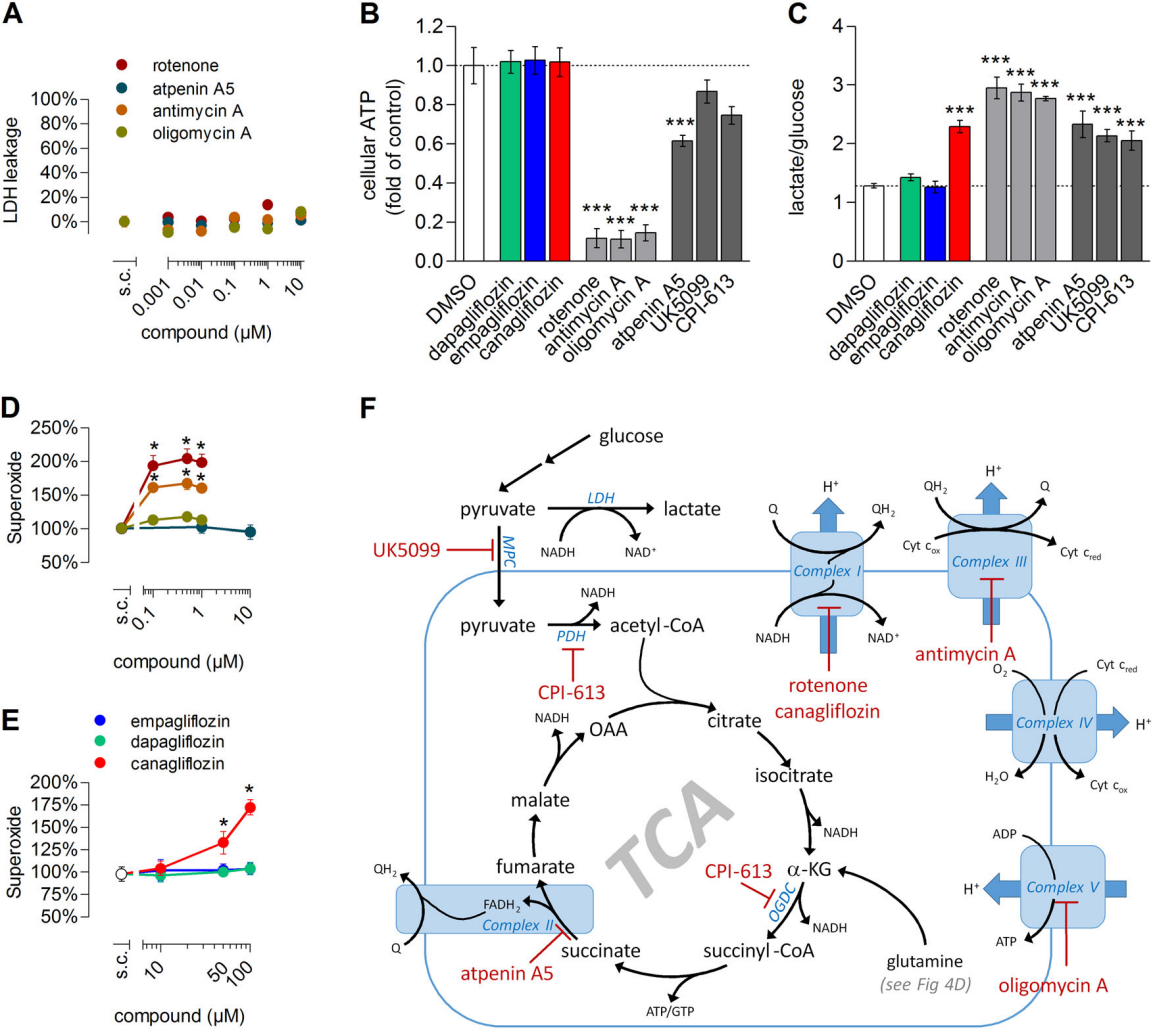
Mitochondrial interference causes anaerobic glycolysis but does not necessarily reduce cellular ATP levels. **a** Cytotoxicity of ETC inhibitors rotenone (complex I), atpenin A5 (complex II), antimycin A (complex III) and oligomycin A (complex V/ATP synthase) on proliferating RPTEC/TERT1. Mean ± SEM, *n* = 3 independent experiments performed in duplicate. **b** Cellular ATP levels and **c** glucose to lactate conversion rates of differentiated RPTEC/TERT1 cells exposed for 24 h to 0.5% DMSO, gliflozins (50 µM), the ETC inhibitors rotenone, antimycin A and oligomycin A (all 1 µM) or compounds interfering with mitochondrial substrate utilization: atpenin A5 (1 µM), UK5099 (200 µM) and CPI-613 (100 µM). Mean ± SEM, *n* = 3 independent experiments performed in duplicate. ***P < 0.001 relative to DMSO assessed by one-way ANOVA with Bonferroni’s post-test. **d**, **e** Superoxide production of differentiated RPTEC/TERT1 after exposure to different ETC inhibitors (**d**) or gliflozins (**e**). Mean ± SEM, *n* = 6 independent experiments performed in quintuplicates. *P < 0.05 relative to solvent control (s.c., 0.5% DMSO) assessed by one-way ANOVA with Dunnett’s post-test. **f** Schematic of glucose metabolism, the TCA cycle and the ETC. Inhibitory sites of compounds used in subsequent experiments are indicated. LDH lactate dehydrogenase, MPC mitochondrial pyruvate carrier, PDH pyruvate dehydrogenase, OGDC α-ketoglutarate dehydrogenase complex, TCA tricarboxylic acid cycle

**Fig. 4:**
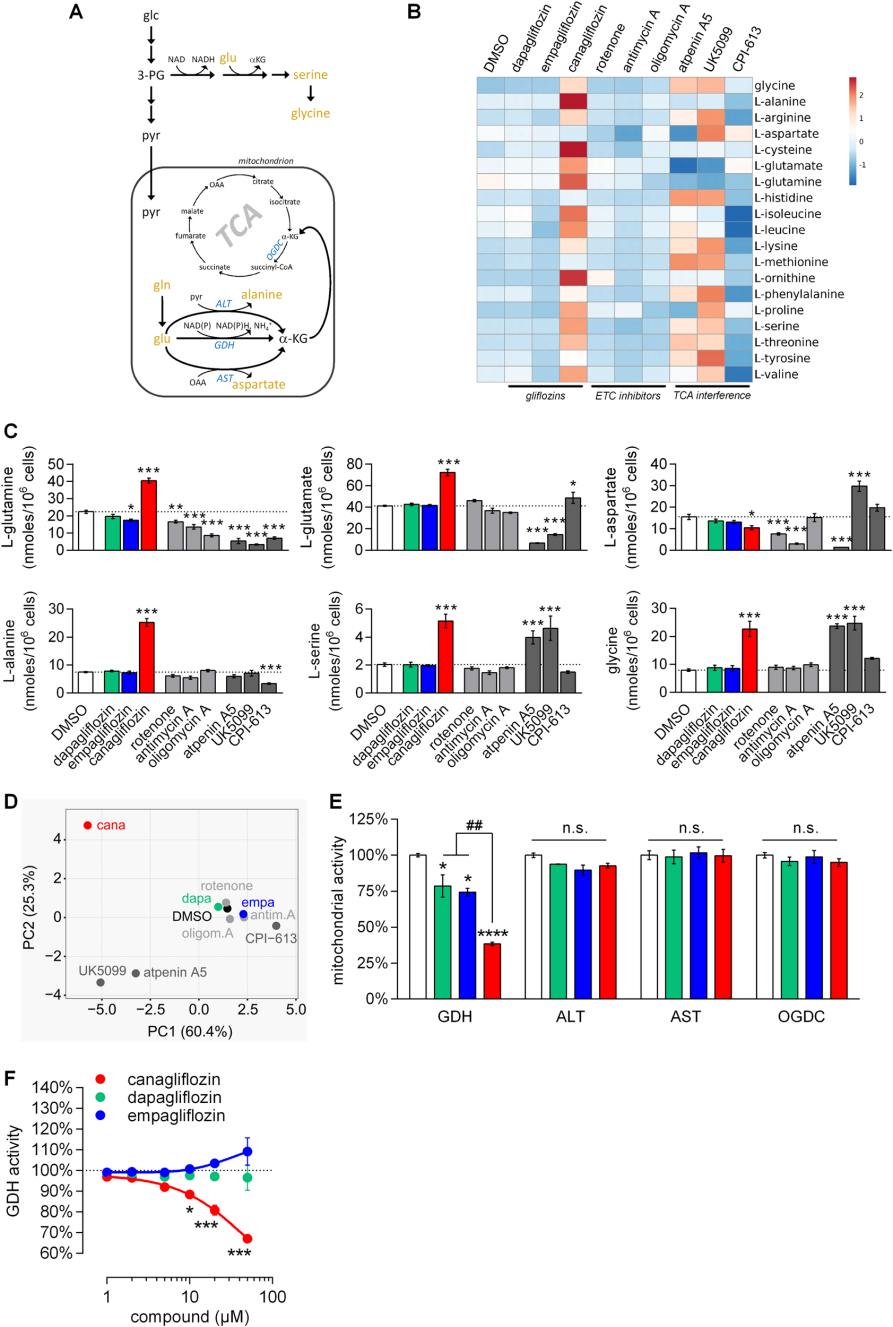
Canagliflozin causes intracellular amino acid accumulation by inhibiting GDH. **a** Schematic of the most prevalent cellular transamination and deamination processes. Glc glucose, 3-PG 3-phosphoglycerate, pyr pyruvate, gln glutamine, glu glutamate, αKG α-ketoglutarate, OAA oxaloacetate, ALT alanine aminotransferase, GDH glutamate dehydrogenase, AST aspartate aminotransferase, OGDC α-ketoglutarate dehydrogenase complex, TCA tricarboxylic acid cycle. **b** The heatmap shows relative intracellular levels of 19 amino acids (AA) in differentiated RPTEC/TERT1 treated for 24 h as indicated. Rows are centered; unit variance scaling is applied to rows. Mean AA concentrations from three independent experiments performed in duplicate were used for calculation. **c** Absolute levels of l-glutamine, l-glutamate, l-aspartate, l-alanine, l-serine and glycine. Mean ± SEM, *n* = 3 independent experiments. *P < 0.05, **P < 0.01, ***P < 0.001 relative to DMSO assessed by one-way ANOVA with Bonferroni’s post-test. **d** Principal component analysis (PCA) plot shows the relationship of the samples based on intracellular AA levels. Unit variance scaling is applied to rows; SVD with imputations is used to calculate principal components and the first two dimensions are displayed. **e** Activity of GDH, ALT, AST and OGDC in mitochondrial preparations from RPTEC/TERT1 cells in the presence of 0.5% DMSO or the different gliflozins (50 µM). Mean ± SEM, *n* = 3 independent experiments. *P < 0.05, ****P < 0.0001; n.s., not significant relative to DMSO. ^##^*P* < 0.01 relative to canagliflozin assessed by one-way ANOVA with Bonferroni’s post-test. **f** Bovine liver GDH activity in the presence of the different gliflozins. Mean ± SEM normalized to 0.5% DMSO, *n *= 3 independent experiments. *P < 0.05, ***P < 0.001 relative to 1 µM of respective compound assessed by one-way ANOVA with Dunnett’s post-test

ETC inhibition is well known to harbor an antiproliferative potential, primarily via reduction of cellular NAD^+^/NADH ratio and thus depleting aspartate and citrate levels required for anabolism^19^. Cytotoxicity, i.e., cell death induction, in contrast, is rarely observed for classical ETC inhibitors under standard culture conditions^20^ and is accompanied by ATP depletion^21^ or reactive oxygen species production^22^. Accordingly, none of the tested ETC inhibitors—rotenone (complex I), atpenin A5 (complex II), antimycin A (complex III) and oligomycin A (complex V)—were cytotoxic in proliferating RPTEC/TERT1 cells (Fig. 3a), despite the use of concentrations ensuring maximal inhibition as indicated by near-complete ATP depletion (Fig. 3b), massive increase in glycolytic rate (Fig. 3c) and—in case of rotenone and antimycin A—pronounced superoxide production (Fig. 3d). Testimony of its effect on complex I, canagliflozin mildly increased cellular superoxide levels (Fig. 3e) but had no effect on cellular ATP levels (Fig. 3b). We thus concluded that canagliflozin must impact on an additional pathway, beyond the inhibition of complex I activity, to explain the observed cytotoxicity. We uncovered this additional pathway by comparing the steady-state metabolic alterations induced by gliflozins with those of established mitochondrial inhibitors (Fig. 3f), i.e., UK5099, targeting mitochondrial pyruvate transporter^23^, and CPI-613, an inhibitor of pyruvate dehydrogenase (PDH) and α-ketoglutarate dehydrogenase (OGDC)^24,25^, in addition to the already introduced ETC inhibitors. Similar to canagliflozin, UK5099 and CPI-613 increased glucose/lactate conversion rates (Fig. 3c), but did not significantly affect ATP levels (Fig. 3b). The latter could suggest that canagliflozin could also be inhibitive at the level of pyruvate import and pyruvate dehydrogenase, in the glutamine to α-ketoglutarate (αKG) conversion or further down in the TCA cycle (Fig. 3f). As amino acids are key metabolites central to different metabolic pathways, e.g., glycolysis or the TCA cycle (Figs. 3f and 4a), we analyzed the gliflozin-induced alterations within the intracellular amino acid (AA) pool of differentiated RPTEC/TERT1 cells. Canagliflozin, atpenin, UK5099 and CPI-613 treatment dramatically altered the cellular amino acid pool, whereas no such alteration was observed with the typical ETC inhibitors or the other two gliflozins (Fig. 4b and Fig. S4). Strikingly, canagliflozin was the only treatment resulting in a pronounced increase in l-glutamate, l-glutamine and l-alanine (Fig. 4b,c). Glutamine is deaminated to glutamate (Fig. 3f), which can be further metabolized to αKG either via transamination, producing other amino acids such as serine, alanine or aspartate, or via deamination, thereby releasing ammonium (Fig. 4a). The αKG produced is fed into the TCA cycle where it is primarily oxidatively metabolized to produce oxaloacetate (OAA) and ATP, or reductively to citrate. The fact that glutamine and glutamate together accounted for more than 50% of the total free amino acids in control cell lysates (Fig. S5) highlight the importance of the latter metabolic route. The reduction of aspartate, the only amino acid being decreased upon canagliflozin exposure, is in line with data obtained for the ETC inhibitors rotenone (complex I) and antimycin A (complex III). Decreasing the NAD^+^/NADH ratio by ETC inhibition reduces OAA and thus the level of its transamination product aspartate. Atpenin A5 truncates the TCA cycle at complex II, thereby causing a characteristic complete depletion of aspartate via depletion of OAA. UK5099, in contrast, cuts off the pyruvate supply to mitochondria, resulting in a concomitant OAA and aspartate accumulation. Both depletion or accumulation of OAA and aspartate resulted in an increased dependence on glutaminolysis for ATP production, as indicated by the severely depleted glutamine and glutamate pools observed (Fig. 4c). In addition, canagliflozin, atpenin A5 and UK5099 exposure increased concentrations of the cytosolic (glycolytic) transamination products serine and glycine (Fig. 4c), most likely as a means to increase αKG levels for oxidation in the TCA cycle (Fig. 4a). However, only canagliflozin raised alanine levels, suggesting an increased activity of alanine aminotransferase (ALT). Overall, quantification of cellular amino acids revealed a strong alteration in cellular amino acid metabolism by canagliflozin, which is distinct from classical ETC inhibitors or UK5099 and atpenin A5, as is shown by the principal component analysis (Fig. 4d), and is suggestive of an impaired utilization of glutamine to yield αKG and other metabolites, and thus an impaired glutamine catabolism.

### Canagliflozin inhibited glutamate dehydrogenase

Indeed, accumulation of glutamine, glutamate, most transamination products of glutamate as well as the striking accumulation of alanine could potentially be explained by reduced deamination of glutamate via specific inhibition of GDH by canagliflozin (Fig. 4b). We thus utilized mitochondrial enriched fractions of RPTEC/TERT1 cells and analyzed the enzymatic activities of all mitochondrial enzymes directly involved in feeding glutamine into the TCA cycle, either in the presence or absence of the three gliflozins. Canagliflozin and to much lower extent dapagliflozin and empagliflozin reduced GDH, but not ALT, aspartate aminotransferase (AST) or α-ketoglutarate dehydrogenase complex (OGDC) activity (Fig. 4e). Moreover, canagliflozin-mediated GDH inhibition was also demonstrated with purified bovine liver GDH, which was not inhibited by the other two gliflozins (Fig. 4f), confirming that the unique canagliflozin-induced cellular amino acid profile likely originated from inhibition of mitochondrial GDH.

### Prevention of mitochondrial pyruvate transport sensitized cells towards canagliflozin

**Fig. 5:**
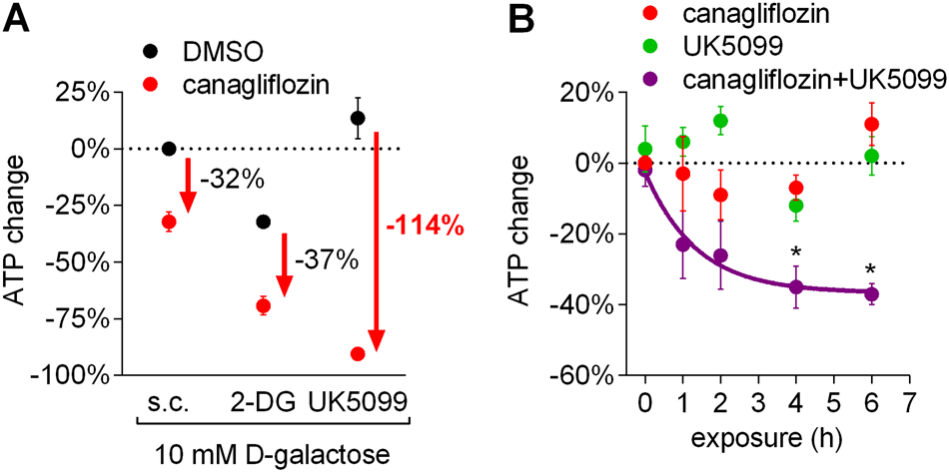
Impaired glycolysis and prevention of mitochondrial pyruvate use sensitizes towards canagliflozin. **a** ATP levels of differentiated RPTEC/TERT1 cells treated for 24 h with DMSO or 50 µM canagliflozin in medium containing 10 mM d-galactose but no glucose with or without 5 mM 2-deoxyglucose (2-DG) or 200 µM UK5099. Mean ± SEM, *n* = 4 independent experiments performed in duplicate. **b** Time course of ATP levels in differentiated RPTEC/TERT1 cells treated with 50 µM canagliflozin, 200 µM UK5099 or both in medium containing 10 mM d-galactose normalized to time matched solvent controls (0.5% DMSO). Mean ± SEM, *n* = 4 independent experiments performed in duplicate. *P < 0.05 relative to 0 h assessed by one-way ANOVA with Dunnett’s post-test

As canagliflozin inhibited ETC complex I and GDH, we determined how specific metabolic settings affect the toxicity of canagliflozin in RPTEC/TERT1 cells. Canagliflozin treatment for 24 h did not result in decreased ATP levels in differentiated cells, suggesting incomplete complex I inhibition with sufficient compensatory glycolytic ATP production. To corroborate this assumption, we exchanged glucose for galactose, thus limiting glycolysis. Under the latter conditions, canagliflozin reduced cellular ATP levels by 32% (Fig. 5a). Addition of 2-deoxyglucose (2-DG), a competitive glycolytic inhibitor, reduced cellular ATP levels by 30%, and in combination with canagliflozin, decreased ATP by an additional 37% down to 30% of control, suggesting that canagliflozin indeed limits mitochondrial but not glycolytic ATP production (Fig. 5a).

Yang et al.^26^ demonstrated that inhibition of mitochondrial pyruvate uptake by UK5099 altered mitochondrial metabolism towards increased glutaminolysis and induced mitochondrial pyruvate synthesis from glutamine via GDH and the TCA cycle. An additional block of GDH activity caused synthetic lethality in their model. Correspondingly, we found diminished glutamine and glutamate levels under UK5099 treatment indicative of elevated glutaminolysis (Fig. 4c). Based on canagliflozin’s potential to inhibit GDH, we hypothesized that combining canagliflozin with UK5099 would strongly impair mitochondrial energy production. Indeed, while UK5099 alone had no effect, the combination with canagliflozin completely depleted cellular ATP levels (Fig. 5a), starting already 1 h after exposure (Fig. 5b). A reduction of ATP levels by combination of UK5099 and canagliflozin was also observed in medium containing 5 mM but not 20 mM d-glucose, demonstrating the importance of aerobic glycolysis to maintain cellular ATP levels under this condition (Fig. S6).

### Combined GDH and ETC inhibition by canagliflozin was antiproliferative

**Fig. 6:**
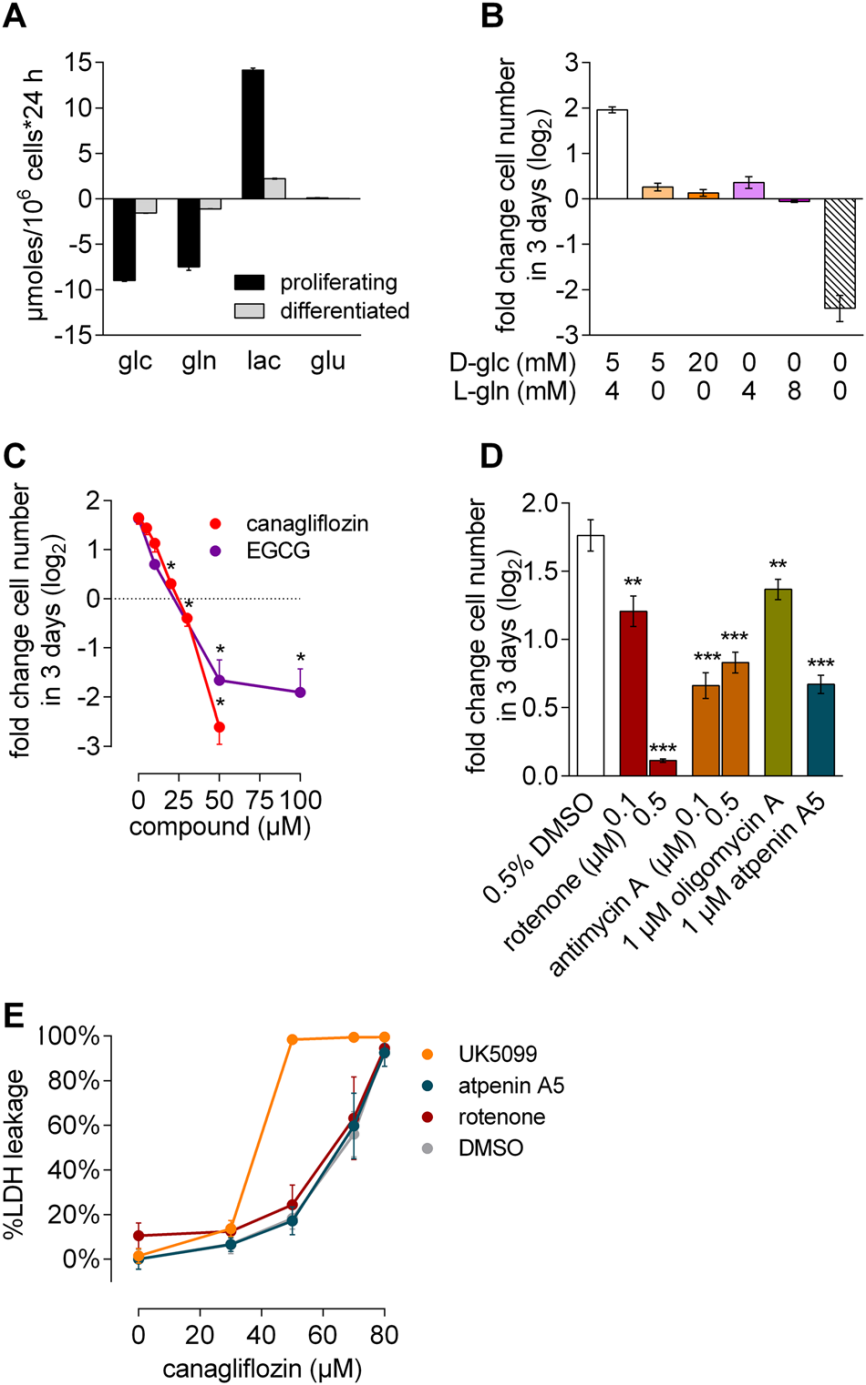
RPTEC/TERT1 cells strongly depend on glutamine and GDH activity for survival and proliferation. **a** Cellular consumption of glucose (glc) and glutamine (gln) and secretion of lactate (lac) and glutamate (glu). Mean ± SEM, *n* = 3 independent experiments performed in duplicate. **b** Proliferation rates of RPTEC/TERT1 cells in medium containing different carbon sources and concentrations. Mean ± SEM of at least three independent experiments performed in triplicate. Proliferation of RPTEC/TERT1 cells exposed to **c** canagliflozin or EGCG or **e** different ETC inhibitors at 20 mM d-glucose, 4 mM l-glutamine and 1 mM pyruvate. Mean ± SEM, *n* = 3 independent experiments performed in duplicate. *P < 0.05, **P < 0.01, ***P < 0.001, relative to 0.5% DMSO assessed by one-way ANOVA with **c** Dunnett’s or **d** Bonferroni’s post-test. **e** Cytotoxicity as quantified by LDH leakage in proliferating RPTEC/TERT1 cells treated for 24 h with different concentrations of canagliflozin in the presence of either 0.5% DMSO, 1 µM rotenone, 1 µM atpenin A5 or 200 µM UK5099. Mean ± SEM, *n* = 3 independent experiments performed in duplicate

The TCA cycle is a critical source for intermediates required for amino acid, fatty acid and steroid synthesis during cell homeostasis but more importantly during cell proliferation and differentiation. Refueling the TCA cycle (anaplerosis) is thus of key importance for proliferating cells, as would be the case, e.g., in the regenerative phase after kidney injury, or during chronic exposure to toxins resulting in continuously high proliferation rates^10^. In the TCA cycle, glutamine is the most important anaplerotic precursor^27^. Beyond increased glutamine catabolism, proliferating cells show high glycolytic rates to support ribose production as well as serine and glycine synthesis required for biosynthetic reactions^28^. Correspondingly, proliferating RPTEC/TERT1 cells showed almost equal consumption of glucose and glutamine, and compared to differentiated cells, overall consumption is increased 5–6 -old (Fig. 6a). Since no net excretion of glutamate was observed, it can be assumed that most of the glutamine consumed is indeed utilized for anabolic reactions. Furthermore, proliferation of RPTEC/TERT1 cells depended on availability of both glucose and glutamine, whereas absence of either resulted in cytostasis (Fig. 6b). In order to characterize the impact of canagliflozin on proliferation, we exposed proliferating cells to canagliflozin in medium containing 4 mM l-glutamine and 20 mM d-glucose to avoid glucose limitation, and quantified cell numbers after 72 h. Canagliflozin exposure resulted in a concentration-dependent reduction of proliferation, whereby 20–30 µM canagliflozin were cytostatic and concentrations >30 µM were cytotoxic (Fig. 6c). Strikingly, the GDH inhibitor epigallocatechin gallate (EGCG)^29^ also caused cytotoxicity, demonstrating that GDH activity is crucial for this cell line during proliferation (Fig. 6c). In contrast, neither dapagliflozin nor empagliflozin had any effect on cell proliferation when tested to up to 100 µM (Fig. S7). Moreover, complete ETC inhibition with rotenone, antimycin A or oligomycin A, and TCA truncation with atpenin A5 significantly reduced proliferation but did not cause cell death during 72 h of incubation (Fig. 6d), supporting the hypothesis that GDH inhibition by canagliflozin is responsible for cell death in RPTEC/TERT1 cells. Final confirmation of this was obtained by co-treating proliferating cells with ETC inhibitors or UK5099 in combination with increasing concentrations of canagliflozin. Addition of rotenone or atpenin A5 did not alter cytotoxicity of canagliflozin, corroborating that the mechanism underlying the observed cytotoxicity is independent of complex I and II activity. Therefore, canagliflozin-mediated toxicity cannot originate from ETC inhibition (Fig. 6e). However, under conditions of increased glutaminolysis induced by UK5099, RPTEC/TERT1 cells were sensitized towards canagliflozin but not towards rotenone-mediated inhibition (Fig. 6e and Fig. S8). With these data we were able to demonstrate that the antiproliferative and cytotoxic activity of canagliflozin in RPTEC/TERT1 cells is mediated mainly by inhibiting GDH-dependent glutamine anaplerosis but not ETC complex I inhibition.

## Discussion

Recent FDA Drug Safety communications warned of gliflozin treatment-associated diabetic ketoacidosis and an increased risk of acute kidney injury primarily in conjunction with canagliflozin but also with dapagliflozin T2DM treatment, specifically relevant for patients with preexisting chronic kidney disease or a history of kidney injury^5,6^. These patients present with a major reduction of physiologically functional nephrons, sustained regenerative proliferation and increased areas of non-functional scar tissue^30^. Concomitantly, the remaining intact nephrons are challenged not only by having to maintain renal functionality but also, under the condition of concurrent T2DM treatment with gliflozins, with an increased in situ (per proximal epithelial cell) concentration of gliflozins, a fact often noted also for many other therapeutics, which can be countered by dosing adjustment^31^. Accordingly, the typical C_max_ for gliflozins calculated from the average T2DM patient may be largely surpassed in patients with preexisting kidney injury, chronic kidney disease^32^ or elderly patients subject to polypharmacy^33–35^. Most of the gliflozin-associated cases of acute kidney injury and ketoacidosis occur under canagliflozin treatment^5,6^, which suggests a compound-specific rather than a class effect.

Despite their capacity for inhibiting SGLT2, gliflozins did not result in glucose limitation in the human proximal tubule epithelial cell line RPTEC/TERT1 in vitro, even at concentrations that were close to or exceeded the C_max_ observed under therapeutic conditions. On the contrary, glucose uptake and utilization was enhanced upon canagliflozin exposure (Fig. S2B), thus suggesting that the observed findings are SGLT independent. By quantification of mitochondrial activities combined with a metabolomics approach, we discovered that at similar concentrations canagliflozin impaired two discrete mitochondrial enzymatic functions, namely GDH and ETC complex I. The latter inhibition and their downstream effects were observed at concentrations of 10–50 µM, thus reflecting the clinical C_max_ (10 µM) and the concentrations (approximately 65 µM) employed in the animal experiments^13^. The canagliflozin-mediated inhibition of complex I activity in cancer cell lines was recently published by Villani et al.^36^ thus corroborating the off-target findings we observed in human renal epithelial cells. Hawley et al.^37^ demonstrated that canagliflozin but not dapagliflozin or empagliflozin activated AMP-activated protein kinase pathway in vivo via mitochondrial dysfunction, independent of its effect on glucose uptake, also supporting our interpretation that the canagliflozin findings we report here are SGLT independent, substance specific and not a class effect.

**Fig. 7:**
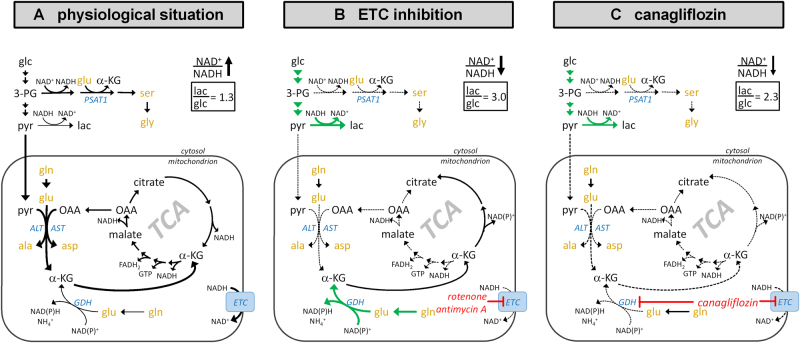
Schematic representation of the most important reactions relevant for glutamine anaplerosis in proliferating cells. **a** Under physiological conditions ETC activity maintains a high NAD+/NADH ratio, allowing for extraction of the glycolytic metabolite 3-PG for serine and glycine synthesis by glutamate transamination. Pyruvate and glutamate can be used by mitochondrial ALT to produce alanine and α-ketoglutarate. OAA levels are high allowing for AST activity. Deamination of glutamate by GDH is dispensable for many cells under this conditions. **b** ETC inhibition reduces NAD^+^/NADH ratio forcing pyruvate to lactate conversion to maintain glycolytic flux and reducing OAA levels. Thereby, 3-PG, pyruvate and OAA become unavailable for transamination and cells depend on GDH activity to secure glutamine anaplerosis. **c** By inhibiting ETC complex I and GDH, canagliflozin interferes with both partially redundant pathways allowing glutamine anaplerosis. This results in glutamine and glutamate accumulation and depletion of TCA metabolites leading to proliferation arrest and finally cytotoxicity

Remarkably, GDH and ETC activity have a well-documented interplay with a direct consequence for glutamine anaplerosis. Under normal physiological conditions, the ETC oxidizes NADH, thus ensuring a high NAD^+^/NADH ratio. This allows for transamination of the three substrates, 3-phosphoglycerate (3-PG), OAA and pyruvate, concomitantly producing αKG for oxidation in the TCA cycle (Fig. 7a). Hence, under conditions of unrestricted glucose supply and ETC function, GDH activity is dispensable for αKG synthesis from glutamate^38,39^. However, when glucose supply and thus availability of transamination substrates is restricted, cells depend on GDH activity for supplying the TCA cycle with carbon from glutamate^40,41^. ETC inhibition, e.g., by rotenone or antimycin A, diminishes the NAD^+^/NADH ratio, forcing pyruvate to lactate conversion (anaerobic glycolysis) and decreasing OAA availability^42^. Hence, similar to glucose restriction under ETC inhibition, substrates for transamination reactions are unavailable, resulting in GDH dependence for glutamate to αKG conversion (Fig. 7b). Indeed, the GDH inhibitor EGCG was reported to exhibit cytotoxicity when combined with either glucose deprivation or UK5099, prohibiting pyruvate transamination^26,39,40^. Consequently, canagliflozin, by inhibiting both the ETC and GDH, reduced the availability of glutamine for the TCA cycle, which resulted in glutamine and glutamate accumulation in differentiated cells and cytotoxicity in proliferating cells, especially as the latter are depleted of anabolic precursors (Fig. 7c).

Our data also showed that RPTEC/TERT1 strongly rely on GDH activity, even in presence of sufficient levels of glucose. In fact, proliferating RPTEC/TERT1 consumed remarkably high amounts of glutamine, i.e., comparable to the consumption of glucose, without secreting glutamate (Fig. 6a). The results obtained with the GDH inhibitor EGCG support the finding that proliferating RPTEC/TERT1 cells are exceptionally sensitive to GDH inhibition (Fig. 6c) when compared to cell types used in other studies, where an effect of EGCG was only observed in combination with inhibitors of glycolysis or mitochondrial pyruvate transport^26,39,40^.

Indeed, proximal tubule epithelial cells under physiological conditions in vivo showed high GDH activity and exhibited very little glycolytic activity, producing OAA and acetyl-coenzyme A for citrate synthesis primarily from glutamine and β-oxidation of fatty acids, respectively^43–45^. In addition to its role in anaplerosis, GDH is also critical for renal regulation of pH homeostasis via the production of ammonium (Fig. 4b and Fig. 7). Indeed, glutaminolysis and GDH activity can be elevated to up to fivefold in the proximal tubule epithelium in response to metabolic acidosis^46^. Thus, the observed GDH inhibition by canagliflozin provides a logical explanation for the reported increased incidence of ketoacidosis observed primarily under canagliflozin treatment^47^. In summary, our results revealed a hitherto unknown mechanism of mitochondrial interference by canagliflozin that provides for a novel explanation of canagliflozin-induced acute kidney injury and ketoacidosis by inhibition of ETC complex I and GDH.

## Materials and methods

### Materials

Canagliflozin, dapagliflozin, empagliflozin, antimycin A, oligomycin A and CPI-613 were from Selleckchem (Munich, Germany). Rotenone, UK5099, thenoyltrifluoroacetone (TTFA) and EGCG were from Sigma-Aldrich (Munich, Germany) and atpenin A5 was purchased from Enzo Life Sciences (Lörrach, Germany).

### Cell culture

RPTEC/TERT1 cells (Evercyte GmbH, Vienna, Austria) were cultured in a 1 to 1 mixture of Dulbecco’s modified Eagle's medium (Life Technologies, Darmstadt, Germany) and Ham’s F-12 nutrient mix (Life Technologies) with 5 mM d-glucose final, supplemented with 2 mM GlutaMAX (Life Technologies), 5 µg/ml insulin (Sigma-Aldrich), 5 µg/ml transferrin (Sigma-Aldrich), 5 ng/ml sodium selenite, 100 U/ml penicillin, 100 µg/ml streptomycin, 10 ng/ml EGF (Sigma-Aldrich) and 36 ng/ml hydrocortisone (Sigma-Aldrich). Cells were subcultured after establishing contact-inhibited monolayer and reseeded at 30% confluence. Proliferating cells were used at day 1 (30–40% confluence). Differentiated cells were cultured for 16 days before use.

### Cell viability and cytotoxicity

Cells were treated with compounds in the presence of 0.5% dimethyl sulfoxide (DMSO), 0.5% DMSO alone (solvent control) or without any treatment (non-treated) as indicated. To quantify cell viability, treatment was exchanged for fresh medium containing 0.5 mg/ml MTT followed by incubation at 37 °C for 45 min. MTT was removed and cells were lyzed in 95% isopropanol and 5% formic acid. Formazan absorption was measured at 550 nm using a Tecan M200Pro microplate reader (Tecan, Männedorf, Switzerland). Values obtained for dead cells (0.1% Triton X-100 treated) were subtracted from all other values and data were expressed as percentage of non-treated cells. Cell death was analyzed separately by quantification of LDH activity in supernatant and cell lysate. Treatment medium was harvested and cells were lyzed in 0.1% Triton X-100/phosphate-buffered saline (PBS). LDH activity was quantified as described in ref.^48^ using a microplate reader. The %LDH leakage was expressed as LDH activity in treatment supernatant divided by the combined activity in supernatant and lysate. LDH leakage obtained for solvent control (0.5% DMSO) was normalized to 0%. Basal LDH leakage was ~15% for proliferating and ~6% for differentiated RPTEC/TERT1.

### Extracellular metabolite quantification

Lactate was quantified exactly as published by Limonciel et al.^49^. Glucose content was quantified using the glucose oxidase (GOD), peroxidase (POD) reaction coupled to 2,2′-azino-bis(3-ethylbenzothiazoline-6-sulphonic acid) (ABTS). Briefly, 10 µl medium samples were mixed with 90 µl reaction solution containing 100 U/ml GOD (Sigma-Aldrich), 0.25 U/ml POD (Sigma-Aldrich) and 1.5 mg/ml ABTS (Sigma-Aldrich) in 0.1 M Na_2_HPO_4_ pH 6.5 followed by incubation for 40 min at room temperature. Absorption at 420 nm was quantified using a plate reader and glucose content was calculated according to a d-glucose calibration curve (0–2 mM). Glutamate was quantified using a modification of the lactate assay, replacing LDH (Sigma-Aldrich) by GDH (Sigma-Aldrich) and using a glutamate calibration curve (0–12.5 mM). Glutamine was deaminated to glutamate prior to quantification. Briefly, samples were acidified by addition of 0.25× volumes of 500 mM acetate pH 5 followed by addition of 0.25× volumes of 5 U/ml glutaminase (Sigma-Aldrich) and incubation for 1 h at 37 °C. Samples were neutralized by addition of 0.25× volumes 1 M Tris base pH 8.3 and glutamate content was quantified as described. Samples incubated in the absence of glutaminase served as background reading for basal glutamate content. A glutamine calibration curve (0–12.5 mM) incubated with glutaminase was used for quantification.

### Seahorse extracellular flux analysis

RPTEC/TERT1 cells were seeded in in Seahorse XFe24 culture plates (Agilent, Waldbronn, Germany) and cultured for 16 days. Cells were analyzed using the Seahorse XF Cell Mito Stress Kit with gliflozins or DMSO being injected via port A as indicated. Cell permeabilization for assessing activity of individual ETC complexes was performed as published by Salabei et al.^50^. In brief, cells were exposed to 50 µM canagliflozin or 0.5% DMSO (subcutaneous (s.c.)) in mannitol and sucrose (MAS) buffer followed by injection of 25 µg/ml digitonin and 1 mM adenosine diphosphate (ADP) in combination with specific substrates for complex I or II (see figure legends) via port A. Coupling of mitochondria was assessed by addition of oligomycin A (1 µM) via port B and subsequent addition of rotenone (1 µM) and antimycin A (1 µM) via port C. Complex IV-dependent respiration was assessed by subsequent addition of tetramethylphenylenediamine (TMPD; 0.5 mM) and ascorbate (2 mM). All concentrations indicated were final concentrations used.

### Preparation of mitochondria-enriched fractions

RPTEC/TERT1 cells (~ 3×10^8^) were harvested by trypsinization and lyzed using a glass-teflon homogenizer. Liquid phase after centrifugation for 10 min at 600 × *g* and 4 °C was collected and centrifuged for 10 min at 11,000 × *g* and 4 °C. Pellet was washed with hypotonic buffer (25 mM potassium phosphate (KPP), 5 mg MgCl_2_, pH 7.2) and centrifuged again. Resulting pellet was dissolved in hypotonic buffer and subjected to three freeze/thaw cycles in liquid N2 prior to enzymatic activity assessments.

### Biochemical quantification of mitochondrial activities

Enzymatic activities were quantified in mitochondrial-enriched fractions. Complex I and II activities were quantified as previously published^51^ with following modifications. Briefly, 25 µl sample (0.7 µg/µl protein) was mixed with 200 µl reaction solution containing 2.5 mg/ml fatty acid bovine serum albumin (BSA; Sigma-Aldrich), 130 µM NADH (Roth, Karlsruhe, Germany), 2 mM KCN (Sigma-Aldrich), 3.6 µM antimycin A, 65 µM ubiquinone 1 (Sigma-Aldrich) and 60 µM 2,6-Dichlorophenol-indophenol (DCIP; Roth) in 25 mM KPP pH 7.5 with or without compound to be tested. The linear phase of the absorption decrease was determined for 3–5 min using a microplate reader. Complex I- independent activity was determined by addition of 5 µM rotenone and was subtracted from all other conditions. Activities were normalized to 0.5% DMSO-treated samples. Complex II activity was determined using same sample volume and readout as for complex I and KPP buffer containing same BSA, KCN, antimycin A, rotenone, ubiquinone 1, DCIP and 20 mM succinate instead of NADH. Complex II-independent activity was determined by addition of 1 µM atpenin A5. ALT and AST activity were quantified using commercial kits (Sigma, #MAK052 and #MAK055) and 70 µg or 12.5 µg total protein, respectively. GDH activity was quantified using 50 µg protein in a 200 µl reaction containing 5 mM αKG, 1.25 mM NADH, 5 µM rotenone, 3.6 µM antimycin A, 10 mM NH_4_Cl and 5 mM MgCl_2_ in KPP buffer pH 7.2 with or without gliflozins. The decrease in NADH absorption at 340 nm was observed for 30 min using a microplate reader. GDH specificity was determined by subtracting activity of a parallel reaction lacking αKG. Purified bovine liver GDH (Sigma, #G2626) was used at 0.01 U per 200 µl reaction. OGDC activity was determined as previously published^52^ with following modifications. Briefly, 100 µl sample (0.25 µg/µl protein) was mixed with 100 µl reaction solution containing 10 mM KPP pH 7.4, 200 µM EDTA, 2 mM CaCl_2_, 2 mM MgCl_2_, 100 µM thiamine pyrophosphate (Sigma-Aldrich), 1 mM dithiothreitol, 150 µM coenzyme A (Sigma-Aldrich), 1.6 mM βNAD (Roth), 5 mM αKG, 5 µM rotenone and 3.6 µM antimycin A. Increases of NADH absorption at 340 nm was monitored for 15 min using a plate reader.

### Superoxide quantification

Superoxide was measured in differentiated RPTEC/TERT1 cells cultured in 96-well plates using a Superoxide detection Kit (Enzo Life Sciences) according to the manufacturer’s instructions using a microplate reader.

### ATP quantification

After treatment as indicated in 96-well plates, differentiated RPTEC/TERT1 cells were washed with PBS and lyzed in 100 µl 50% ice-cold methanol for 10 min at −20 °C. Samples were diluted 1:5 with H_2_O and 10 µl samples were mixed with 90 µl ATP determination kit reagent (Thermo Fisher) and incubated for 7 min prior reading of luminescence using a microplate reader. ATP levels of exposed cells were normalized to 0.5% DMSO-treated cells.

### Cellular AA quantification

RPTEC/TERT1 cells were seeded, reached confluency at 6 days after seeding and were then differentiated for additional 10 days in 6 cm dishes (yielding 6×10^6^ cells) followed by treatment as indicated for 24 h. Cells were washed with PBS and scraped on ice in 1.4 ml 50% (V/V) ice-cold methanol/water. Samples were transferred to clean 1.5 ml tubes and shaken for 30 min at 4 °C followed by centrifugation for 10 min at 16,000 × *g* at 4 °C. Liquid phase was collected and evaporated using a vacuum concentrator followed by solubilization in 150 µl 2% 5-sulfosalicylic acid. AAs were quantified using a Sykam S433 AA analyzer (Sykam, Fürstenfeldbruck, Germany)^53^. Briefly, AAs were separated by high-performance liquid chromatography and subsequent post-column derivatization with ninhydrin. Samples were directly injected in a volume of 100 µl. Chromatography was performed using a lithium-based anion exchange column loaded with spherical polystyrene resin (7 µm diameter, 10% crosslinks, cat. no. 5125022). Elution was performed using buffers with increasing pH and ion strength (pH 2.9 to pH 12; buffer concentration 0.12 M to 0.45 M), supported by a temperature gradient. Absorbance of the reaction products was quantified at 440 nm (intermediate product; quantifies cysteine and proline) or 570 nm (quantifies all other AAs). AA concentrations were determined relative to a reference standard using the area under the peak method in the ChromStar 7 software (SCPA, Weyhe-Leehste, Germany).

### Proliferation assays

Cells were plated at 6×10^4^ per cm² in 24-well plates and allowed to adhere overnight. Cells were treated in 950 µl culture medium for 3 days. At the time of treatment, cells on a satellite plate were fixed with 4% paraformaldehyde to determine baseline cell number. After 3 days, cells were washed with PBS, fixed and Hoechst 33342 stained. Number of nuclei was determined using a CellInsight automated microscope (Thermo Fisher).

### Statistical analysis

Unless otherwise indicated data are presented as mean ± SEM. Sample size (*n*) indicates the number of independent experiments performed. Statistical significance was determined using GraphPad Prism Version 5.04, statistical tests as indicated in the figure legends. Principal component analysis and heatmap visualization was carried out using ClustVis^54^.

## Electronic supplementary material

Supplementary Information
