# Supplementary material for: Canagliflozin mediated dual inhibition of mitochondrial glutamate dehydrogenase and complex I: an off-target adverse effect
Source: Cell Death Dis. 2018 Feb 14;9(2):226. doi: 10.1038/s41419-018-0273-y (PMC5833677; doi:10.1038/s41419-018-0273-y)
Supplement: Supplementary file 1 — Supplementary Information [file 41419_2018_273_MOESM1_ESM.docx]

Canagliflozin mediated dual inhibition of mitochondrial glutamate dehydrogenase and complex I: an off-target adverse effect

Philipp F. Secker^1^, Sascha Beneke^1^, Nadja Schlichenmaier^1^, Johannes Delp^2^, Simon Gutbier^2^, Marcel Leist^2^ and Daniel R. Dietrich^1^

^1^ Human and Environmental Toxicology, University of Konstanz, 78457 Konstanz, Germany

^2^ in-vitro Toxicology and Biomedicine, University of Konstanz, 78457 Konstanz, Germany

Running title: Canagliflozin inhibits glutamine anaplerosis

Corresponding author: Dr. Daniel R. Dietrich, Human and Environmental Toxicology, P-O- Box 622: University of Konstanz, 78457 Konstanz, Germany

Tel: +49 7531 883518; E-mail: daniel.dietrich@uni-konstanz.de

SUPPLEMENTARY DATA

Contains Supplementary Figures S1-S8 including legends


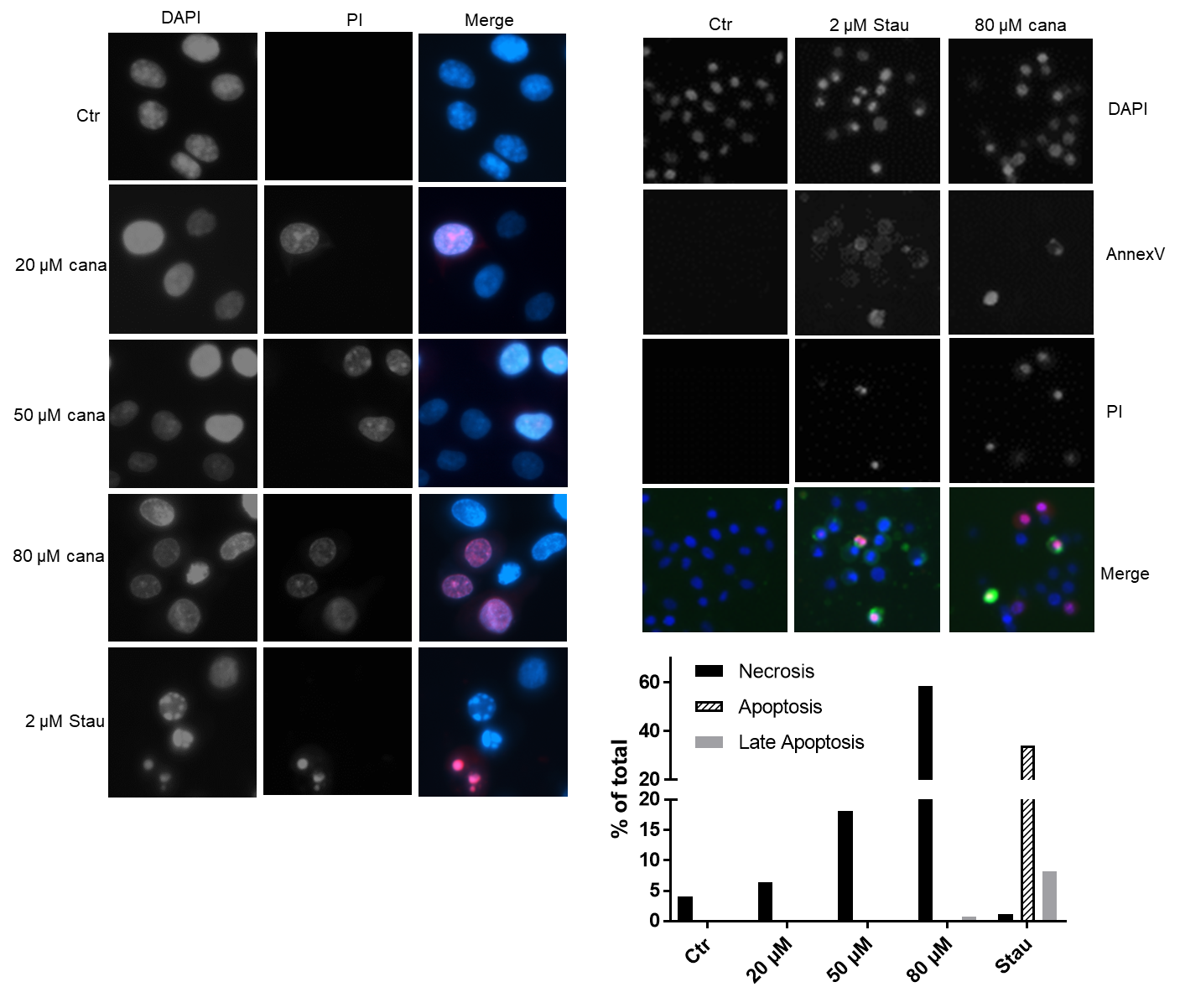


**Figure S1A. Canagliflozin treatment induces necrosis in RPTEC/TERT1 cells**

Proliferating RPTEC/TERT1 cells were treated as indicated in Supplementary Methods (see below) and analyzed for nuclear morphology by DAPI and PI staining (left panel) and annexin V staining (right panel). Figures are representative for observations made in the respective experiments. At least 100 cells were counted for each treatment and the percentage of detected cell death pathway was calculated from nuclear morphology experiments (bar graph). Whereas staurosporine induces apoptosis as visualized by formation of apoptotic bodies and positive annexin V staining without PI signals, only necrosis is detectable in canagliflozin treated cells as judged by nuclear morphology, DAPI and PI double-positive nuclei and annexin V signals in few necrotic cells only. AnnexV: annexin V; cana: canagliflozin; Ctr: control; PI: propidium iodide; Stau: staurosporine.


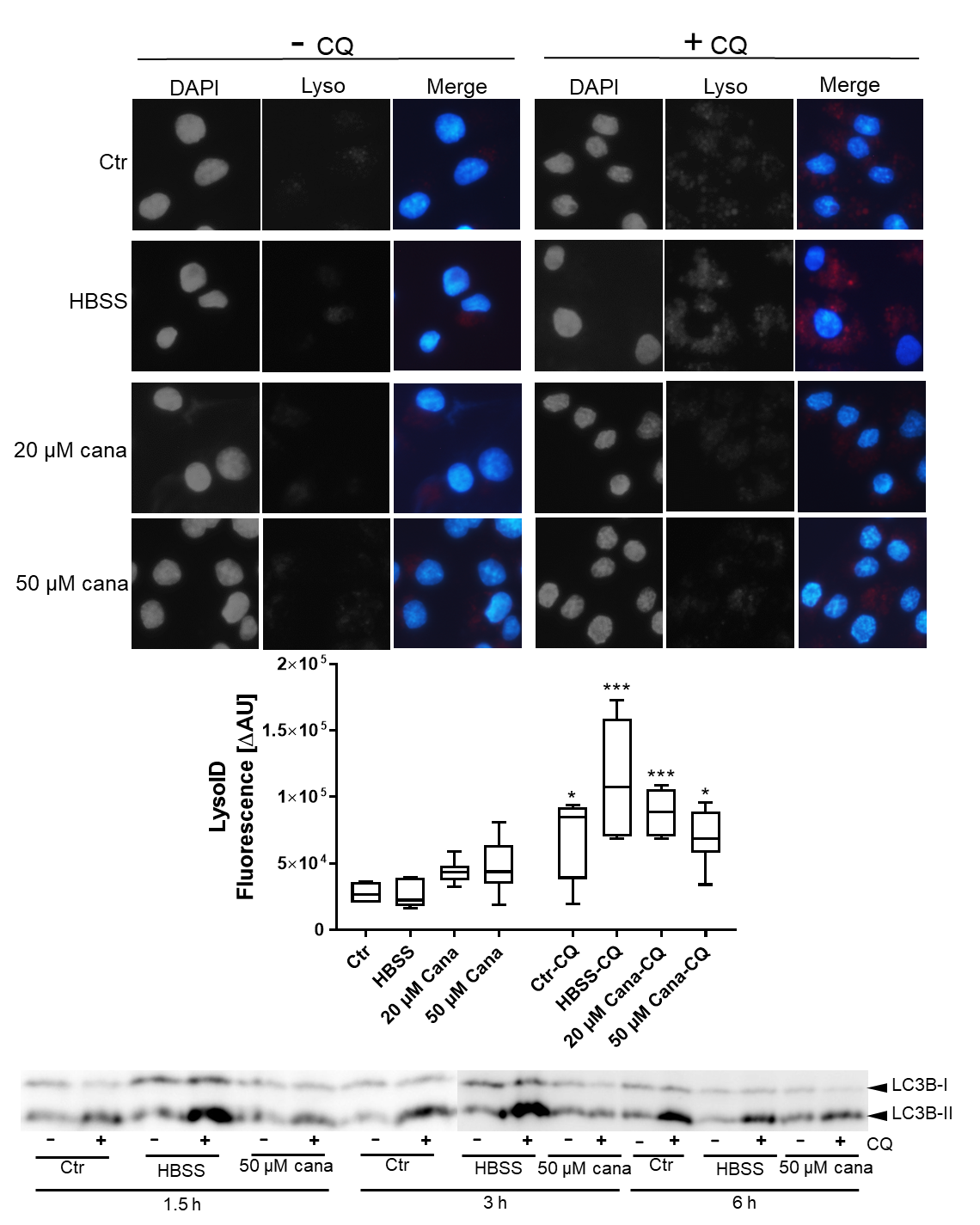


**Figure S1B. Canagliflozin does not induce autophagy in RPTEC/TERT1 cells**

Proliferating RPTEC/TERT1 cells were treated as indicated in Supplementary Methods (see below) and analyzed for autophagy by staining for lysosomal compartments with LysoID. Upper panel pictures are representative for observations made in the respective experiments. LysoID fluorescence intensities from 4-8 pictures and two wells were measured using Fiji and normalized to background and cell number (middle panel). Canagliflozin does not significantly induce an autophagic response compared to control (one-way ANOVA with Dunnett’s post test). * indicates significant differences between samples with and without CQ by one-sided t-test. Whisker-Box plots show 5-95^th^ percentiles and the median of respective data. Data are supported by results from immunoblots showing LC3B-I to LC3B-II conversion (lower panel).

CQ: chloroquine; Ctr: solvent control; cana: canagliflozin; Lyso: LysoID.


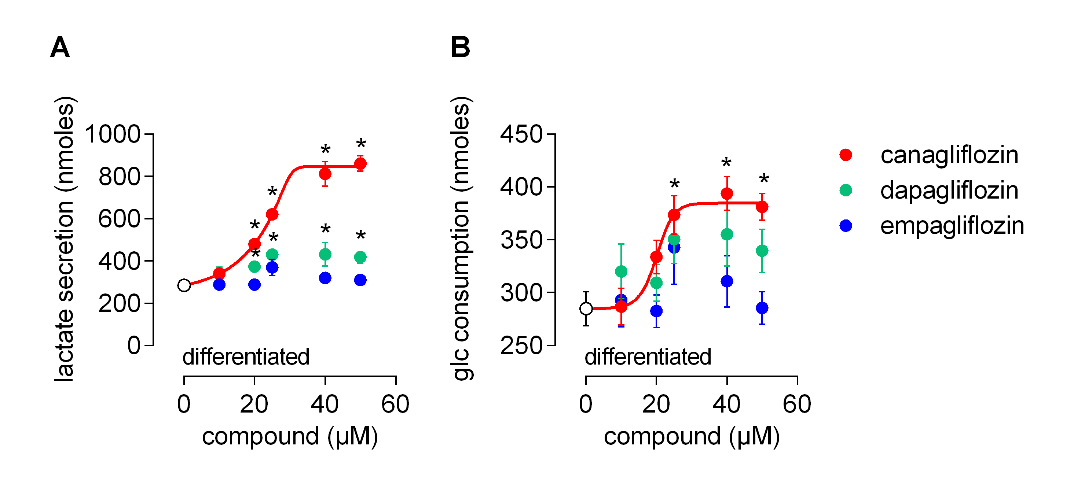


**Figure S2. Canagliflozin strongly elevates lactate secretion and glucose consumption of differentiated RPTEC/TERT1 cells.** (A) Lactate secretion and (B) glucose consumption of differentiated RPTEC/TERT1 cells cultured in 96-well plates was quantified after 24 h exposure to the different gliflozins. Data are mean ± SEM of at least three independent experiments. **P* < 0.05 relative to solvent control (s.c., 0.5% DMSO). ANOVA with Dunnett’s post-test.


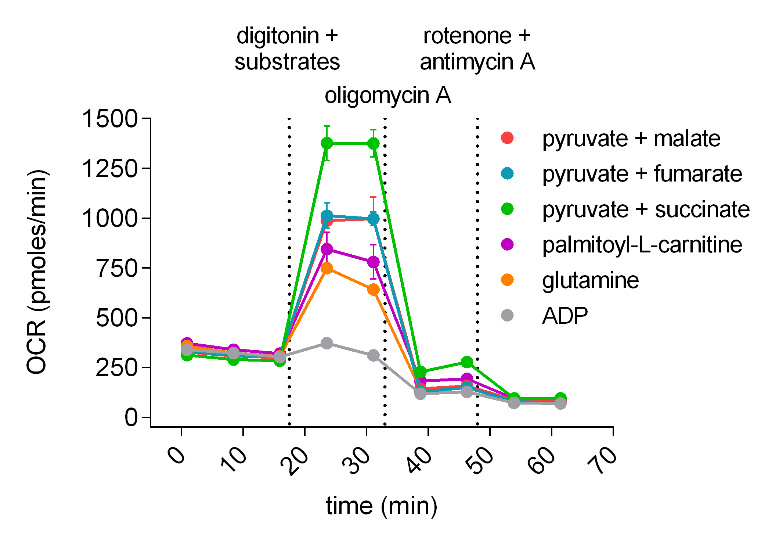


**Figure S3. Digitonin-permeabilized RPTEC/TERT1 cells remain coupled mitochondria.** Differentiated RPTEC/TERT1 cells were permeabilized using 25 µg/ml digitonin in MAS buffer (70 mM sucrose, 220 mM mannitol, 10 mM KH_2_PO_4_, 5 mM MgCl_2_, 2 mM HEPES, 1 mM EGTA, pH 7.2 (KOH)) containing different substrates plus ADP or ADP alone followed by addition of 1 µM oligomycin A and finally 1 µM rotenone and 1 µM antimycin A. Data represents mean ± SD of a representative experiment performed in duplicates.

**
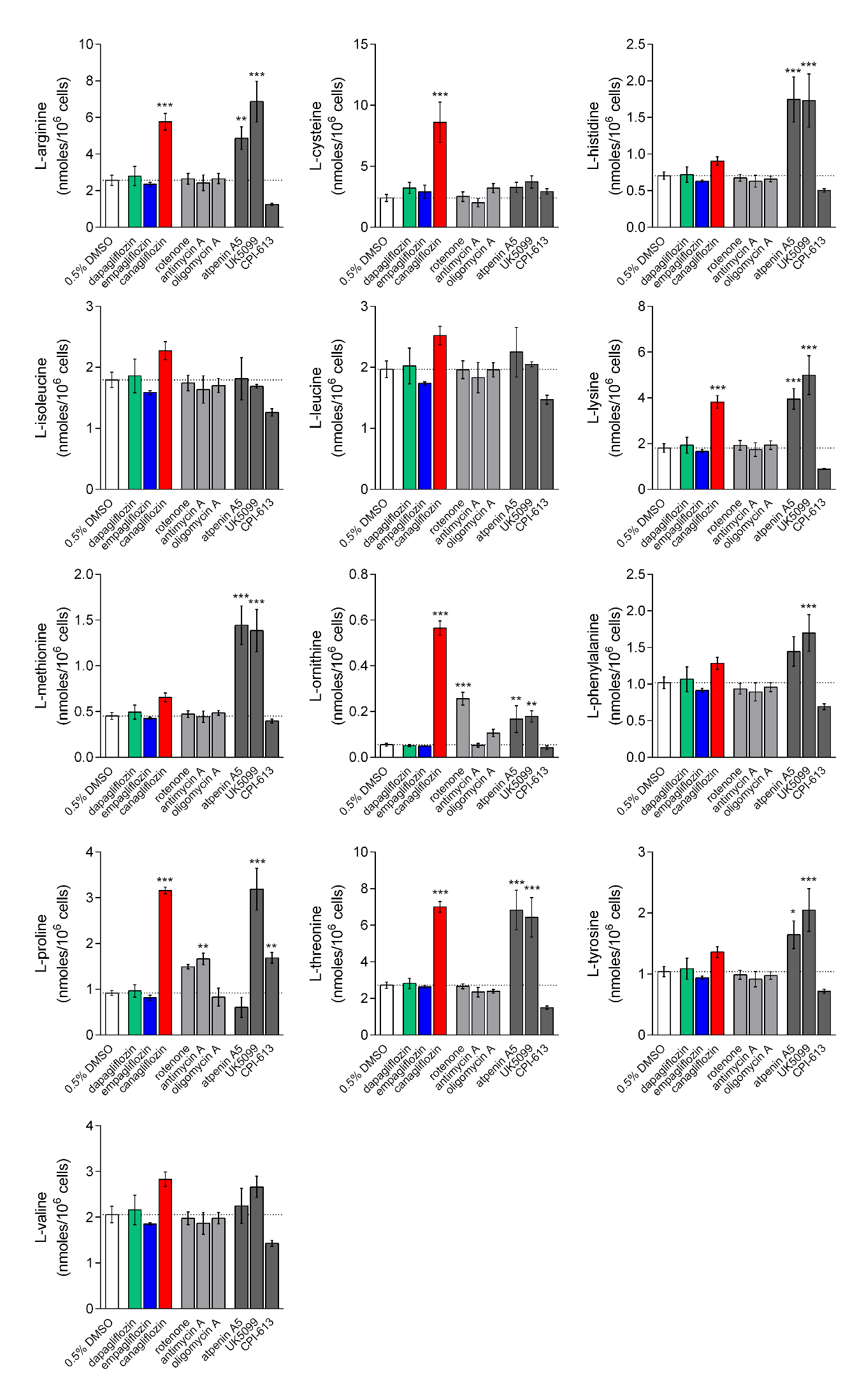
**

**Figure S4. Amino acid levels of differentiated RPTEC/TERT1 cells treated for 24 h as indicated.** A total of 6*10^6^ cells were treated for 24 h as indicated followed by amino acid extraction and quantification. Mean ± SEM, n = 3 independent experiments. **P* < 0.05, ***P* < 0.01, ****P* < 0.001 relative to DMSO. ANOVA with Bonferroni’s post-test.


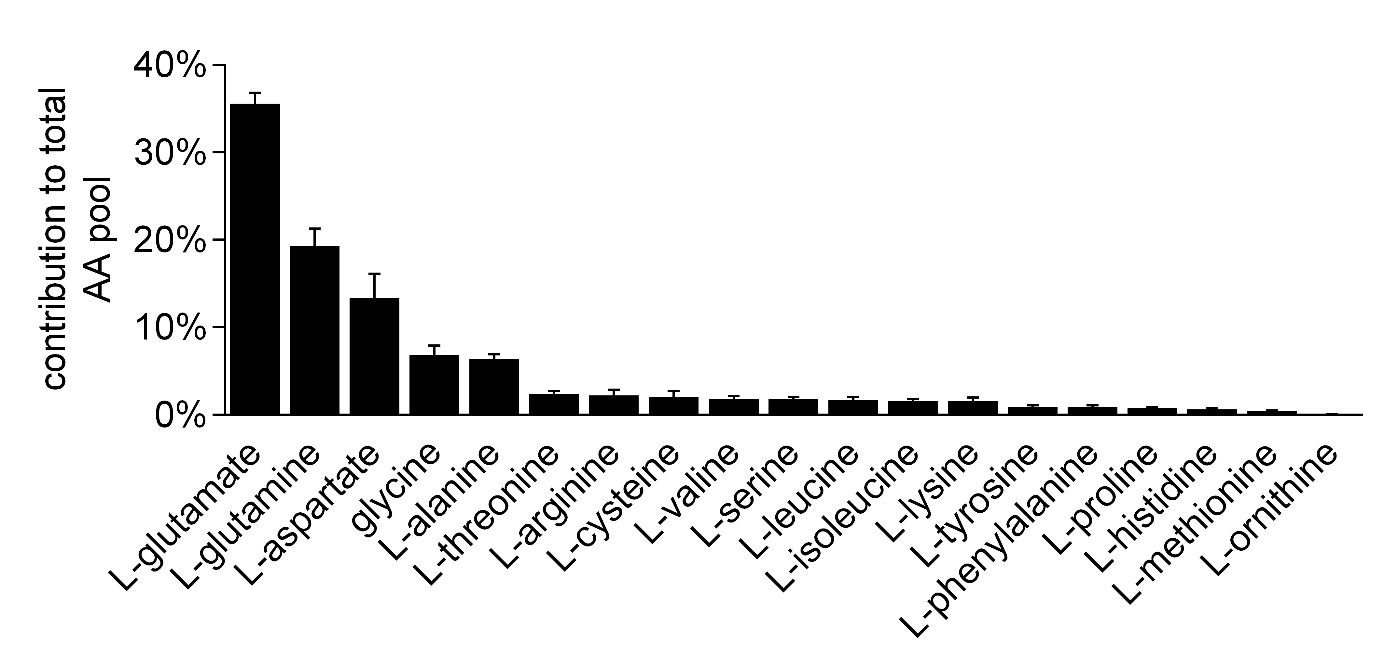


**Figure S5. Relative contribution of all 19 quantified amino acids to the intracellular amino acid pool of RPTEC/TERT1 cells.** Amino acids of 6*10^6^ differentiated RPTEC/TERT1 cells were extracted and the 19 amino acids displayed here were quantified. The total cellular amino acid concentration was 116 µmoles/10^6^ cells. The molar fraction of the individual amino acids was divided by the total concentration and is given as percent. Data presents mean ± SEM, n = 3 independent experiments.

**
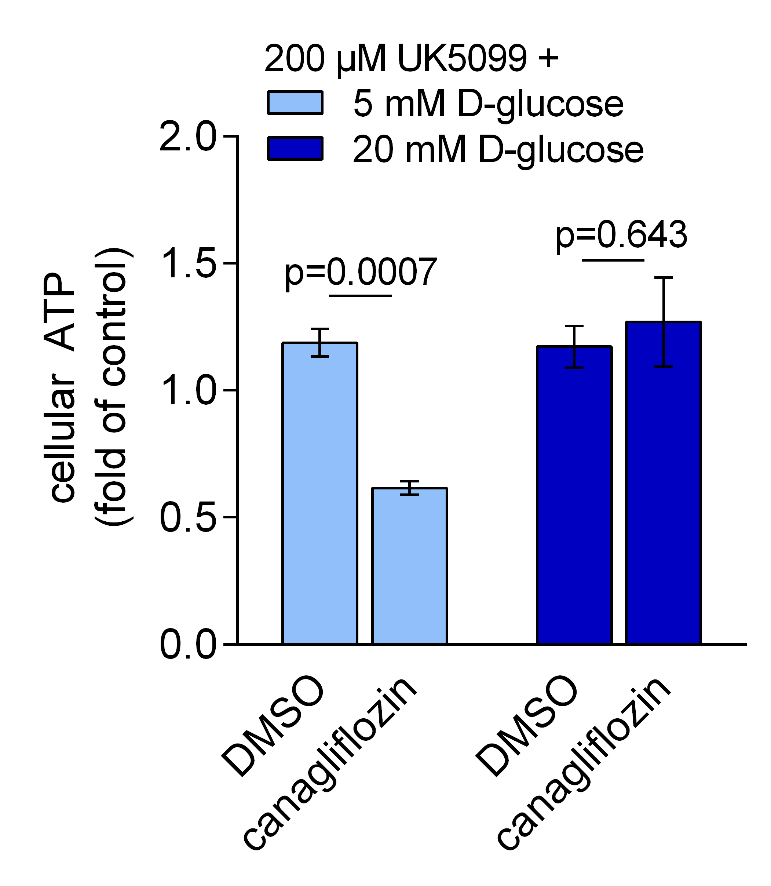
**

**Figure S6. RPTEC/TERT1 cells require high glycolytic flux to maintain ATP under canagliflozin and UK5099 treatment.** Cellular ATP levels of differentiated RPTEC/TERT1 treated for 24 h with 200 µM UK5099 with or without 50 µM canagliflozin in medium containing either 5 mM or 20 mM D-glucose. Data normalized to treatment with 0.5% DMSO only. Mean ± SEM, n = 4 independent experiments. P values were determined using unpaired t test.


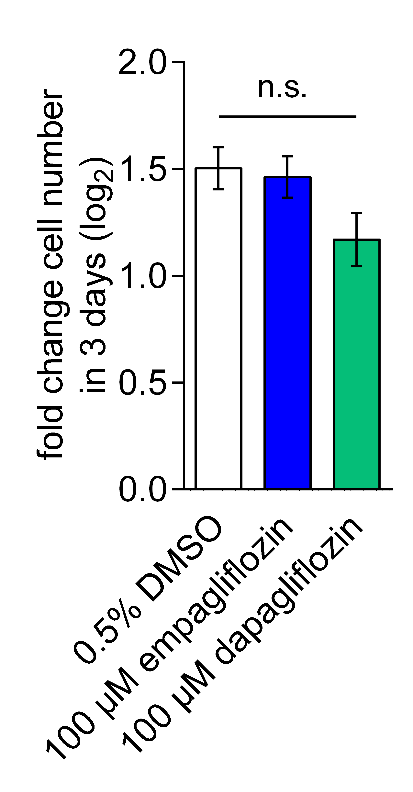


**Figure S7. Dapagliflozin and empagliflozin do not impair RPTEC/TERT1 proliferation.** Proliferating cells were treated with 100 µM empagliflozin or dapagliflozin in medium containing 20 mM D-glucose, 4 mM L-glutamine and 1 mM pyruvate for 3 days followed by quantification of cell number. Mean ± SEM, n = 3 independent experiments. ANOVA with Bonferroni’s post-test.


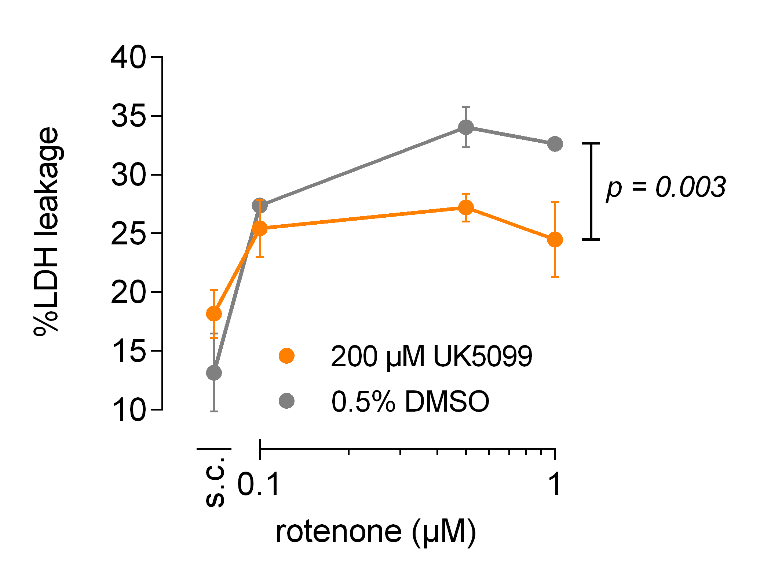


**Figure S8. Prevention of mitochondrial pyruvate transport does not sensitize to ETC inhibition.** LDH leakage of proliferating RPTEC/TERT1 treated with rotenone in presence or absence of 200 µM UK5099 for 48 h. Mean ± SEM, n = 3 independent experiments. P values were determined using two-way ANOVA with Bonferroni’s post-test.

**Supplementary Methods**

Cell death determination:

Cells were treated with compounds in presence of 0.5% DMSO or 0.5% DMSO alone (solvent control) for 24 h at indicated concentrations. As apoptosis control, cells were treated with 2 µM staurosporine (Enzo Life Sciences). Subsequently, cells were incubated in fresh medium containing 2.5 µM Hoechst 33342 and 1 µg/ml propidium iodide (PI) for 10 min in the dark at room temperature for nuclear analysis or stained using the FITC Annexin V Apoptosis Detection Kit with PI (BioLegend) following the manufacturer’s instructions. Briefly, cells were stained with Binding Buffer containing Annexin V (1:100) and 200 µg/ml PI for 15 min in the dark at room temperature. Afterwards, cells were counterstained with Binding Buffer containing 2.5 µM Hoechst 33342 for 10 min at room temperature in the dark. Three different regions each of at least two wells were imaged using a Zeiss Axiovert 200 M inverted epifluorescence microscope. The respective percentage of cells showing apoptosis (nuclear fragmentation, DAPI positive), late apoptosis (nuclear fragmentation, DAPI and PI double positive), or necrosis (DAPI and PI double positive without nuclear fragmentation) was calculated, respectively.

Autophagy analysis:

Cells were treated for 6 h with compounds in presence of 0.5% DMSO, 0.5% DMSO alone (solvent control) or HBSS no glucose as indicated. Chloroquine was added to a concentration of 100 µM to show accumulation of lysosomes by impairing acidification as positive control. For immunohistochemistry cells were stained using the LysoID Red detection Kit (Enzo life Sciences) according to the manufacturer’s instructions. Briefly, cells were stained with Assay Buffer containing LysoID Red Detection Reagent (1:1000) and Hoechst 33342 (1:1000) for 30 min at 37 °C in the dark and imaged using a Zeiss Axiovert 200 M inverted epifluorescence microscope with fixed exposure time for LysoID detection. Fluorescence intensity of LysoID was measured using Fiji and normalized to background signal and cell number.
